# The Spread of Lumpy Skin Disease Virus across Southeast Asia: Insights from Surveillance

**DOI:** 10.1155/2023/3972359

**Published:** 2023-05-19

**Authors:** Lillian Wilhelm, Michael P. Ward

**Affiliations:** Sydney School of Veterinary Science, The University of Sydney, Camden, Australia

## Abstract

Lumpy skin disease (LSD) is a notifiable, transboundary disease, causing substantial economic and welfare impacts in cattle. Prior to October 2020, LSD had not been reported in Southeast Asia; however, on 5 October 2020, Vietnam reported the first case in the region. This study aimed to investigate the initial spread of LSD virus (LSDV) in cattle across Southeast Asia between October 2020 and October 2021. LSD outbreak data were accessed from the World Organisation for Animal Health (WOAH) World Animal Health Information System (WAHIS) database and analysed to investigate this spread via epidemic curves, disease maps, clustering, and descriptive statistics. During the epidemic period, 866 LSD outbreaks were reported from six Southeast Asian countries, consisting of 1,758,923 susceptible cattle, 93,465 cases, 5,936 deaths, and 1,117 cattle culled. Analysis revealed a propagated epidemic throughout Southeast Asia, with four major peaks in case numbers across Thailand and Vietnam. Three clusters of reported outbreaks were identified, and Thailand was found to be the epicentre of the outbreak in the region, which could reflect reporting bias and underreporting from other countries in Southeast Asia. High morbidity and mortality rates were reported, particularly in Thailand, Vietnam, and Cambodia, likely reflective of infection in a naïve population and lack of an effective vaccination program. These findings are in contrast to what has generally been described in other parts of the world. Furthermore, studies should examine the risk factors associated with high morbidity and mortality rates in this region. A greater understanding of LSD epidemiology in Southeast Asia will assist farmers and governments to implement effective control and prevention strategies that reduce the spread of disease to other regions and the potentially devastating impacts of LSD.

## 1. Introduction

Lumpy skin disease (LSD) is a notifiable, transboundary disease, causing substantial economic and welfare impacts in livestock. LSD is caused by the lumpy skin disease virus (LSDV), belonging to the family *Poxviridae* and genus *Capripoxvirus* [1]. LSD primarily affects cattle, water buffalo, and wild ruminants; however, few reports of infections in wildlife have been noted [2]. All ages and breeds are susceptible to the disease, but infection is most commonly reported—and is most severe—in young cattle, underweight cattle, and those in peak lactation or immunocompromised [3]. Whilst disease mortality is usually low (1–3%), morbidity rates are high, averaging 5–45% [2, 4]. The condition is characterised by circumscribed skin nodules 2–6 cm in diameter, generally located on the neck, legs, tail, and back [5]. Clinically affected animals also commonly present with pyrexia, enlarged lymph nodes, depression, reduced milk yield, and abortion [2]. LSDV can remain viable in the environment for up to 35 days, with the main sources of LSDV being necrotic skin lesions, scabs, and blood [6]. Transmission can occur via blood-sucking arthropods, such as mosquitoes and *Stomoxys* spp., contaminated feed and water, and bodily secretions [7]. Increased outbreaks and case numbers occur in summer and during wet periods when vector species are abundant, suggesting viral spread is linked primarily to vector transmission [7, 8].

Endemic initially in Africa, LSD has spread in parts of Europe via the movement of infected animals and vector transmission, amplified by seasonally linked outbreaks [7, 9]. LSDV environmental persistence and various modes of transmission create challenges for control and prevention programs worldwide. Despite this, the widespread use of live-attenuated vaccines during the Balkans outbreak (2015–2017) proved successful in controlling this epidemic [10]. In addition, early outbreak detection, stamping out (culling affected and cattle suspected of being infected), and cattle movement restrictions have assisted in control programs worldwide [10]. Although these control measures have been implemented in endemic areas, LSDV has recently spread to Asia, with reports from China, India, Bangladesh, and Nepal [11, 12]. Subsequently, the first reported outbreak of LSD in Southeast Asia occurred in Vietnam (Huu Lung District, Lang Son Province) in October 2020 [13]. During 2020-2021, the disease spread to five other Southeast Asian countries: Cambodia, Laos, Malaysia, Myanmar, and Thailand. Due to the recent nature of LSD occurrence in Southeast Asia, current knowledge regarding its status and outbreak trends within the region is sparse. Given the extent of this outbreak and lack of knowledge surrounding LSD in Southeast Asia, it is critical to investigate LSD epidemiological patterns in this region. Together with welfare concerns, data from previous outbreaks indicate LSD to be a substantial financial burden to producers because of reduced milk yield, trade restrictions, and treatment and prevention costs [2, 14]. Therefore, investigating trends in LSD epidemiology in Southeast Asia could assist in reducing further spread and mitigate the consequential impacts of the disease.

The aim of this study was to investigate and map the spread of lumpy skin disease in cattle across Southeast Asia between October 2020 and October 2021, during its early spread phase. An analysis of outbreaks was undertaken to determine patterns in case numbers, locations, timing of outbreaks, and epidemic progression throughout the region. This investigation was based on data obtained from the World Organisation for Animal Health (WOAH) World Animal Health Information System (WAHIS) database.

## 2. Materials and Methods

### 2.1. Data Collection and Management

The locations and dates of reported LSD outbreaks throughout Southeast Asia were sourced from the WOAH. Immediate notifications and follow-up reports were downloaded from the Animal Disease Events collection publicly available at the WAHIS interface (https://wahis.woah.org/#/home; last accessed on 15/04/2022). This interface was accessed each month from the end of the study period (October 2021) until April 2022. The reports that were present in the database at that time were considered to be the final study dataset. LSD outbreaks reported in the WAHIS interface were selected if they occurred in a Southeast Asian country (Brunei, Myanmar, Cambodia, Timor-Leste, Indonesia, Laos, Malaysia, the Philippines, Singapore, Thailand, and Vietnam) between 1 October 2020 and 1 October 2021. Reported cases of LSD were diagnosed by authorities in each country based on clinical signs and nucleic acid detection (PCR assays), as well as necropsies (in Myanmar and Thailand). No additional details on diagnostic procedures were documented in the data that was available. From each WAHIS report, the following information was extracted: report date, outbreak start and end dates (if applicable), outbreak location (latitude/longitude and by name), unit type (village or farm), and number of susceptible animals, cases, deaths, and culled. Reports were filtered to include only cattle; reports of LSD in other *Bovidae* (Buffalo, *Capricornis sumatraensis, Bos frontalis*, and *Bos javanicus*) were excluded due to the small number of reports (37) and low case numbers (115). The data collected were imported into Excel 16.0 (Microsoft, Redman WA), and reports were combined into datasets (by country of origin) for further analysis. Error checking was performed: for each data entry, logical values were checked, and manual checking against the original reports from the WAHIS database was also undertaken.

### 2.2. Data Analysis

Epidemic day was calculated by assigning day 1 to the first reported case in the region (5 October, 2020), and then each report date was allocated a number relative to this baseline date.

The daily number of cases and outbreak reports for each country were calculated and plotted for the outbreak period, on a normal and a log scale, to produce epidemic curves (Microsoft Excel 16.0). The locations (latitude and longitude) of reported outbreaks were mapped; this was achieved using a shape file of Southeast Asia (DIVA-GIS, geographic coordinate system (GCS) WGS 1984) within a geographic information system (ArcGIS v10.7. ESRI, Redlands, CA). Data points for each reported outbreak were created by using the reported latitude and longitude values. Based on the day each outbreak was reported to occur, a colour ramp (epidemic day green to red [days 1–361]) was used to visualise the epidemic spread of LSD throughout Southeast Asia. The mean centres and one standard deviation directional ellipses (unweighted and weighted by epidemic day and number of cases) were calculated. These visualisation tools were overlayed on the point map of reported outbreaks to describe the progression of the LSD epidemic in Southeast Asia. The weighting of mean centres and directional ellipses used either the epidemic day at each location or the number of cases reported at each location. Thus, locations at which LSD was reported later or more cases were reported influenced the estimated mean centres and directional ellipses more. In this way, whether outbreak time or intensity influenced the distribution of the epidemic could be investigated. In addition to case locations by epidemic day, a map was created based on reported case numbers using a colour ramp (case numbers green to red [0–11520] to visualise the spatial distribution of the number of reported cases in each outbreak.

A retrospective space-time analysis was conducted (SaTScan v9.6, https://www.satscan.org/) to identify clusters present within the WAHIS reported data. The discrete Poisson (population at-risk) model was utilised, which assumes the number of reported cases at each location is Poisson-distributed, i.e., the expected number of cases at each location is proportional to the population size [15]. Therefore, in this analysis, the numerator was the reported number of cases at each location, and the denominator was the number of susceptible cattle at the same location in the same report submitted to WAHIS. These data were scanned for clusters of locations with high attack rates. The maximum spatial cluster size was arbitrarily set to 20% of the population at-risk and the maximum temporal cluster size to 50% of the study period to identify clusters of interest. Identified clusters were interpreted based on the ratio of expected to observed cases, and the statistical significance was evaluated by log likelihood ratios using a Monte-Carlo simulation with 999 iterations. The statistically significant clusters that were identified were mapped (ArcGIS v10.7. ESRI, Redlands, CA) based on the cluster centre (longitude, latitude) and radius (km).

Descriptive analysis was undertaken using Excel 16.0. Morbidity (number of cases ÷ susceptible animals), mortality (number of deaths ÷ susceptible animals), and the culled rate (number of culled ÷ susceptible animals) estimates were calculated as proportions for each report submitted to WAHIS. “Culled” was interpreted as cattle that were culled by authorities or farmers and disposed of due to infection. Analyses for each country also included the number of outbreaks, susceptible numbers, cases, deaths, and culled, as well as the mean, median, and interquartile range (IQR) of the number of cases and epidemic days.

## 3. Results

Between October 1, 2020, and October 1, 2021, 866 LSD outbreaks were reported from Southeast Asian countries to the WAHIS database (Tables 1 and 2). These reports detailed a total of 1,758,923 susceptible cattle, 93,465 cases, 5,936 deaths, and 1,117 cattle culled (Tables 1 and 3). Outbreaks were first reported from Vietnam (5 October 2020) and Myanmar (9 November 2020), and subsequently from Thailand (29 March 2021), and then finally from Malaysia (10 May 2021), Laos (22 May 2021), and Cambodia (26 May 2021).

**Figure 1:**
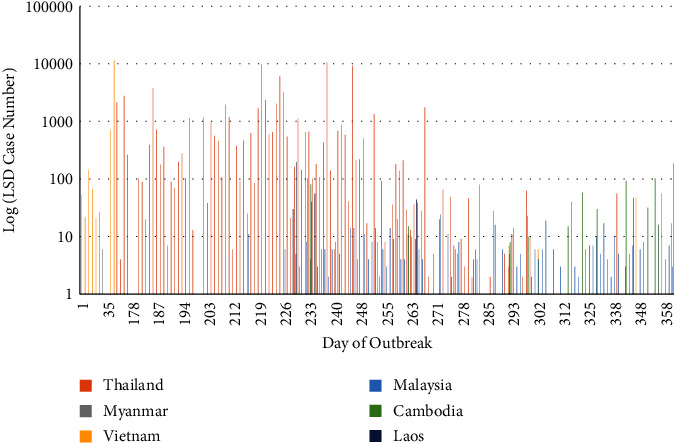
Number of lumpy skin disease (LSD) cases (log scale) reported from Thailand, Malaysia, Cambodia, Vietnam, Laos and Myanmar to the World Organisation for Animal Health (WOAH) World Animal Health Information System (WAHIS; https://wahis.woah.org/#/home) from 1 October 2020 to 1 October 2021.

**Figure 2:**
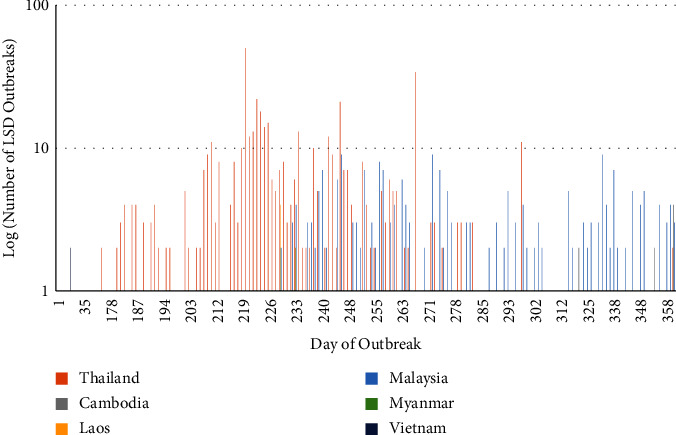
Number of lumpy skin disease (LSD) reports (log scale) from Thailand, Malaysia, Cambodia, Vietnam, Laos, and Myanmar to the World Organisation for Animal Health (WOAH) World Animal Health Information System (WAHIS; https://wahis.woah.org/#/home) from 1 October 2020 to 1 October 2021.

**Figure 3:**
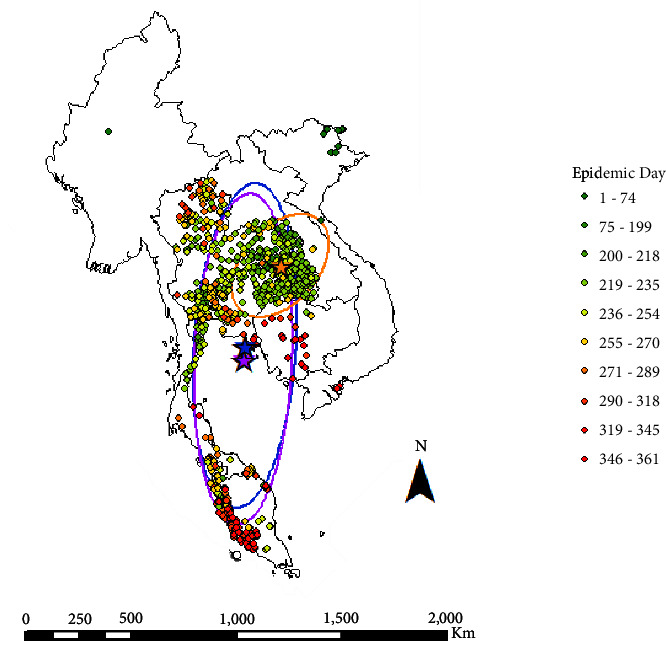
Distribution of lumpy skin disease outbreak sites in Southeast Asia between 1 October 2020 and 1 October 2021. Sites are shaded green to red by epidemic day (1–361). Day 1 = 5 October 2020; day 361 = 1 October 2021. Directional ellipses (1 SD) are overlayed; unweighted (blue) and weighted by cases (orange) and epidemic day (purple). The mean centres indicated by stars; unweighted (blue) and weighted by cases (orange) and epidemic day (purple). Data was extracted from the World Organisation for Animal Health (https://wahis.woah.org/#/home).

Since the number of cases per report was not constant, both cases and reports were plotted to better understand the epidemic. The reported outbreaks across Southeast Asia illustrate a propagated epidemic, with four major peaks in case numbers (Figures 1 and 2). The first peak occurred on day 74 in Vietnam, and the other three peaks occurred on days 220, 238, and 246 in Thailand (Figure 1). The epidemic curves showed Thailand to have experienced an LSD epidemic with concentrated periods of outbreaks and cases, whilst the other countries (Cambodia and Malaysia) experienced more prolonged outbreaks with fewer cases that occurred over a longer period of time (Figures 1 and 2). The majority of the outbreaks (*n* = 533), susceptible cattle (*n* = 1,738,566), cases (*n* = 78,968), and deaths (*n* = 5,874) occurred in Thailand, the epicentre of the Southeast Asia outbreak (Tables 1–3; Figure 3). Secondary to Thailand, Vietnam experienced the next highest case numbers (*n* = 12,703), followed by Cambodia (*n* = 824). Country-specific epidemic curves are shown in Supplementary figures A and B. As expected, all mean centres calculated (unweighted, case, and epidemic day weighted) were situated in Thailand, with the cases centre northeast and the unweighted and epidemic day centres situated close together and to the south in Thailand (Figure 3). The direction of spread during the study period was north to south with a 3.8° rotation. When weighted by epidemic day (1–361), the direction of spread was very similar (2.3° rotation) to the unweighted analysis, but when weighted by number of outbreak cases reported (0–11520), there was a noticeable northeast shift (41.1° rotation), demonstrating the greater impact of LSD in areas of eastern Thailand during the earlier months of this epidemic (Figure 3).

The median outbreak day followed a similar pattern as to LSD spread throughout Southeast Asia (Table 2). Myanmar only reported one outbreak during the Southeast Asia epidemic, and Laos reported nine outbreaks. The epidemics in Laos and Myanmar had few cases, and shortly after, they officially marked their country's outbreak as resolved, with no further cases since the initial occurrence was reported. The majority of cases in Vietnam occurred on one day of the epidemic (74), followed by a substantial period of time without reports of disease. Subsequently, on day 293 of the epidemic period, cases re-emerged, with a slight increase in reported case numbers towards the end of the study period. Malaysia and Cambodia reported LSD outbreaks later in the epidemic, with case numbers continuing to rise towards the end of the study period. Outbreaks in Thailand began on day 156 of the epidemic, and whilst outbreaks continued to be reported throughout the epidemic period, the majority of cases occurred within 100 days of Thailand's first reported case.

**Figure 4:**
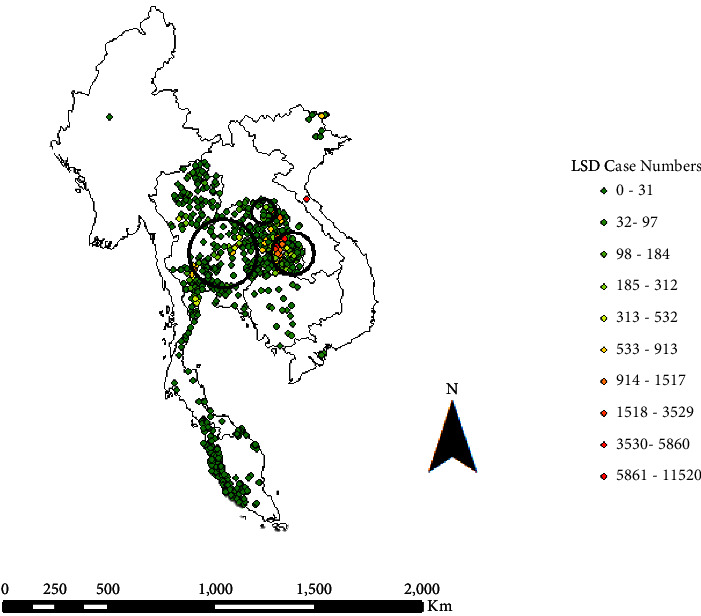
Lumpy skin disease case numbers in Southeast Asia from 1 October 2020 to 1 October 2021. Outbreaks are shaded green to red by case number (0–11520). Primary (13 May to 30 June 2021; east), secondary (21 April to 5 July 2021; west) and tertiary (17 April to 13 May 2021; central) spatiotemporal clusters are overlayed, with observed to expected case numbers being 9.57, 4.91, and 11.06, respectively. The data was extracted from the World Organisation for Animal Health (https://wahis.woah.org/#/home).

Three major clusters were identified by spatial data analysis and considered to be statistically significant. The primary cluster was centred in east Thailand but included Laos and occurred between 13 May and 30 June 2021. Within this cluster (radius 125 km), there was an observed-to-expected ratio of cases of 9.57 (*P* < 0.001) (Figure 4). The secondary cluster occurred in central Thailand and occurred over a longer time period, between 21 April 21 and 5 July 2021. The radius of this cluster was greater (195 km), with an observed-to-expected ratio of 4.91 (*P* < 0.001) (Figure 4). The tertiary and smallest cluster occurred in northeast Thailand between 17 April and 13 May 2021. Within this cluster (radius 67 km), there was an observed-to-expected ratio of cases of 11.06 (*P* < 0.001) (Figure 4).

Overall, across all six countries, case morbidity was 20.9%, case mortality was 2.7%, and the culled rate was 0.7% (Table 1). Morbidity was highest in Thailand (37.1%) and Cambodia (22.4%), followed by Malaysia (19.5%), Laos (19.1%), Vietnam (18.1%), and Myanmar (9.5%) (Table 1). Mortality was highest in Vietnam (7.7%) and Thailand (7.3%), whilst the other countries had a mortality rate of less than 1.1% (Table 1). The culled rate was highest in Vietnam (4.4%), followed by Thailand (0.03%), with the other countries reporting no cattle culled (Table 1).

## 4. Discussion

This study provides an overview of the epidemiology of LSDV throughout Southeast Asia in 2020-2021, via descriptive statistics, epidemic curves, disease mapping, and cluster analysis. Compared to what is reported in the literature, we estimated high morbidity and mortality rates in Southeast Asia and identified Thailand as the epicentre of this regional epidemic.

The data analysed in this investigation were limited to what was accessible and reported from each country to the WOAH WAHIS database, presenting a key limitation to this study. As this investigation relied solely on each country reporting information about their LSD outbreaks, there is potential for reporting bias. Countries with frequent reporting likely provided a more accurate description of their epidemic, whereas countries with infrequent reporting, a lack of resources, poor surveillance of LSD cases, and differing policies contribute to the lack of data accuracy. Whilst reporting should be standardised and therefore equivalent across countries, there is no guarantee that this will occur. However, the analysis of reported cases provided some important insights into the spread of LSDV in Southeast Asia during the initial incursion phase. Considering the very large number of cases reported to the WOAH WAHIS database and that LSD was a novel disease syndrome during 2020-2021, the biases inherent in such surveillance data were unlikely to substantially affect the overall conclusions made. Different outbreaks can also be grouped together and reported as single outbreaks. This cluster of reporting creates barriers when inferring farm dynamics and epidemiology trends from data, and cluster detection identified areas where there were reports with higher attack rates than expected (Poisson model), rather than areas with a higher incidence. Unidentified cases throughout Southeast Asia were also likely, with a risk of under-detection due to subtle clinical signs, subclinical cases, or unobservant farmers. Estimates of morbidity and mortality might depend on when outbreaks were investigated and the timeliness of reporting and follow-up reports. We made the assumption that outbreak investigation protocols during this early period of the LSD epidemic in Southeast Asia were broadly consistent, so that systematic bias was minimised. It must be noted that data from outbreaks in Southeast Asia were collated until 15 April 2022 and therefore might differ from any current revisions to the data in the WOAH WAHIS database. Data were updated throughout the study with revisions made periodically to the dataset.

Most cases of LSD were reported from Thailand and Vietnam. Whilst more disease could have been experienced in these countries, it is likely that more effective surveillance systems resulted in more outbreaks being reported. During this early phase of the LSD epidemic in Southeast Asia, methods of control were likely similar across the region, a region in which livestock management systems are typically based on smallholders. Implementation of disease control and prevention, such as the use of vaccines, once a disease becomes endemic will result in changes to the spatiotemporal distribution of disease occurrence. Although more LSD outbreaks were reported from Vietnam and Thailand, the overall consistent spread of LSD through Southeast Asia in 2020-2021 suggests that reporting of the disease varied by country. More uniform surveillance would enable a more detailed epidemiological analysis of this epidemic.

Overall, we estimated a mean case morbidity rate in Southeast Asia, based on reports, of 20.9%, with a very large range from 0.001 to 100%. Across the world, LSD morbidity rates can vary depending on a range of both within and between country factors, with an average rate between 5 and 45% reported [4]. Studies investigating previous epidemics in Asia report similar morbidity rates ranging from 0.3 to 30% [12, 16–18]. In contrast, morbidity rates reported from other areas of the world have been lower than in the current study, such as 8.6% in Iraq [19] and 8.7% in Greece [20]. In this study, the highest morbidity rates were estimated for Thailand and Cambodia, 37.1% and 22.4%, respectively. Without further information detailing the management systems in place for affected premises in this study, it is difficult to establish the definite cause for the high morbidity rates. Literature suggests morbidity rates can be influenced by multifactorial issues regarding host susceptibility, the environment, and the pathogen. Cattle breed, host immunological status, vector population, climate, husbandry, management conditions, and LSDV strain are key aspects to consider [2]. With regard to management conditions, studies have found smallholder operations with smaller herds and fewer cattle to have a statistically significant greater morbidity risk, compared to more intensive operations [5]. With smallholder management systems dominating in southeast Asia, this might explain the higher morbidity rates estimated.

The mean mortality rate across Southeast Asia estimated in this study was 2.7%. This estimate is higher than average mortality rates reported in other countries yet remains less than the upper end of the expected range (<10%) [4]. Across other Asian countries, such as India, Hong Kong, Bangladesh, and China, mortality rates of 0 to 0.9% have been reported [12, 16–18]. In other regions in the world, the mortality rate is often low, generally reported to be <3% [19, 20]. The mortality rates investigated in this study showed Vietnam to have the highest (7.7%), closely followed by Thailand (7.3%). These high rates could be due to many reasons, potentially including sampling bias. These two countries had the highest reporting rates for susceptible animals and cases, making their reported outbreak scale much larger than other countries. Other possible reasons for elevated mortality rates include the agro-ecological zones and husbandry management systems affected [21], the entirely naïve cattle populations [22], and lack of access to vaccines. A phylogenetic analysis has indicated LSDV isolates in Thailand to be 99.83% similar to the strains from mainland China, Hong Kong territory, and Vietnam in a potential virulent vaccine-recombinant strain [23]. According to reports submitted to the WOAH WAHIS database, Thailand coordinated a multifaceted control and prevention response to its LSD epidemics. This included movement restrictions on animals in affected provinces, decreased bovine importation, public awareness campaigns to support early detection, distribution of insecticides, and establishing containment zones within a 50 km radius of outbreaks [24]. Early approval of LSD vaccinations by the Thai Food and Drug Administration, followed by a vaccine plan, allowed the first vaccines to arrive in Thailand on 29 May 2021, two months after the first case report in the country [24]. Although the vaccination program was promptly initiated, LSDV spread had already occurred prior to implementation. It is unclear how the vaccination schedule was executed or if all regions of Thailand had available access, adequate resources, and education to vaccinate their cattle appropriately. The role of vaccination in Southeast Asia requires investigation.

## 5. Conclusions

This investigation found LSD in Southeast Asia to have generally high morbidity and mortality rates in the epidemics that occurred between 1 October 2020 and 1 October 2021. The lack of an effective vaccine program and the naivety of the Southeast Asian cattle population likely contributed to these high estimates; however, further research on specific risk factors influencing morbidity and mortality rates in Southeast Asia is warranted. Whilst outbreaks were reported from six Southeast Asian countries, Thailand was identified as the epicentre of this regional epidemic during the study period. Vector abundance, strain virulence and transmissibility, management, control, and prevention factors likely contributed to the predominance in this region. To assist in mitigating further spread of disease, particularly to the Asia-Pacific and Oceania regions, future studies should focus on risk factors for LSDV transmission, high morbidity and mortality rates as well as the efficacy of control and prevention strategies in Southeast Asia.

## Figures and Tables

**Table 1:** Summary statistics of lumpy skin disease (LSD) cases, morbidity, and mortality rates in Thailand, Malaysia, Cambodia, Vietnam, Laos, and Myanmar, reported to the World Organisation for Animal Health (WOAH) World Animal Health Information System (WAHIS) from 1 October 2020 to 1 October 2021.

Parameters	Thailand	Malaysia	Cambodia	Vietnam	Laos	Myanmar	Average	Total
Number of reports	553	267	21	15^*∗*^	9	1	144.33	866
Number of susceptible cattle	1738566	11271	3907	3098	2018	63	293153.83	1758923
Number of cases	78968	595	824	12703	369	6	15577.5	93465
Number of deaths	5874	1	13	48	0	0	989.33	5936
Number culled	20	0	0	1097	0	0	186.17	1117
Mean morbidity (%)	37.1	19.5	22.4	18.1	19.1	9.52	20.95	—
Minimum morbidity (%)	0.001	0.20	13.33	3.76	14.91	9.52	6.95	—
Maximum morbidity (%)	100	100	41.67	48	20	9.52	53.20	—
Mean mortality (%)	7.3	0.07	1.1	7.7	0	0	2.68	—
Minimum mortality (%)	0	0	0	0	0	0	0	—
Maximum mortality (%)	100	20	6.67	53.57	0	0	30.04	—
Mean culled (%)	0.03	0	0	4.4	0	0	0.73	—
Minimum culled (%)	0	0	0	0	0	0	0	—
Maximum culled (%)	14.08	0	0	15.09	0	0	4.86	—

The total number of reports (*n* = 866), number of susceptible cattle (*n* = 1758923), number of cases (*n* = 93465), number of deaths (*n* = 5936), and number of culled (*n* = 1117) were reported for each country. The morbidity and mortality were calculated as number of cases ÷ number of susceptible cattle and number of deaths ÷ number of susceptible cattle, respectively. The proportion culled was calculated as number culled ÷ number of susceptible cattle. An average was calculated and reported across all countries (*n* = 6). The data was extracted from the WOAH WAHIS (https://wahis.woah.org/#/home). ^*∗*^Vietnam only officially reported 15 outbreaks; however, 2 outbreaks contain suboutbreaks that account for the majority of the cases (outbreak 84273 had 205 suboutbreaks, accounting for 11,520 of the cases and outbreak 84272 has 45 suboutbreaks, accounting for 722 cases).

**Table 2:** Summary statistics of lumpy skin disease (LSD) reports (*n* = 866) from Thailand, Malaysia, Cambodia, Vietnam, Laos, and Myanmar to the World Organisation for Animal Health (WOAH) World Animal Health Information System (WAHIS) from 1 October 2020 to 1 October 2021.

Parameters	Thailand	Malaysia	Cambodia	Vietnam	Laos	Myanmar	Average
Number of reports	553	267	21	15	9	1	144.33
Minimum epidemic day	1	1	1	1	1	1	1
1^st^ quartile	221	253	300	12	230	35	175.17
Mean	234.4	286.5	321	103.5	235.3	35	205.62
Median	227	275	332	22	230	35	186.83
3^rd^ quartile	249	325	355	183.5	234	35	230.25
Maximum epidemic day	205	145	127	358	31	1	144.5
IQR	28	72	55	171.5	4	0	55.08

Day 1 is assigned to the start date for each group's epidemic. An average was calculated and reported across all countries (*n* = 6). The data was extracted from the WOAH WAHIS (https://wahis.woah.org/#/home).

**Table 3:** Summary statistics of lumpy skin disease (LSD) cases in Thailand, Malaysia, Cambodia, Vietnam, Laos, and Myanmar, reported to the World Organisation for Animal Health (WOAH) World Animal Health Information System (WAHIS) from 1 October 2020 to 1 October 2021.

Parameters	Thailand	Malaysia	Cambodia	Vietnam	Laos	Myanmar	Average
Number of susceptible cattle	1738566	11271	3907	3098	2018	63	293153.83
Number of cases	78968	595	824	12703	369	6	15577.5
Minimum cases in single report	0	1	6	1	19	6	5.5
1^st^ quartile	3	1	15	7.5	28	6	10.08
Mean	147.60	2.23	39.24	846.87	41	6	180.49
Median	13	1	25	27	42	6	19
3^rd^ quartile	74	2	70	58.5	51	6	43.58
Maximum cases in single report	10317	17	103	11520	78	6	3673.5
IQR	71	1	55	51	23	0	33.5

The number of susceptible animals (*n* = 1758923) and number of cases (*n* = 93465) were reported for each country. An average was calculated and reported across all countries (*n* = 6). The data was extracted from the WOAH WAHIS (https://wahis.woah.org/#/home).

## Data Availability

The data are available via the World Animal Health Information System, https://wahis.woah.org/#/home.

## Supplementary Materials

Supplementary Materials Supplementary figure A: number of lumpy skin disease (LSD) cases (log scale) reported from each of Thailand, Malaysia, Cambodia, Vietnam, Laos, and Myanmar to the World Organisation for Animal Health (WOAH) World Animal Health Information System (WAHIS; https://wahis.woah.org/#/home), 1 October 2020 to 1 October 2021. Supplementary figure B: number of lumpy skin disease (LSD) outbreaks (log scale) reported from each of Thailand, Malaysia, Cambodia, Vietnam, Laos, and Myanmar to the World Organisation for Animal Health (WOAH) World Animal Health Information System (WAHIS; https://wahis.woah.org/#/home), from 1 October 2020 to 1 October 2021.
